# The Interplay between Nevi and Melanoma Predisposition Unravels Nevi-Related and Nevi-Resistant Familial Melanoma

**DOI:** 10.3390/genes12071077

**Published:** 2021-07-16

**Authors:** Stefania Pellegrini, Lisa Elefanti, Luigi Dall’Olmo, Chiara Menin

**Affiliations:** 1 Department of Surgery, Oncology and Gastroenterology (DISCOG), University of Padua, 35128 Padua, Italy; stefania.pellegrini.1@studenti.unipd.it (S.P.); luigi.dallolmo@unipd.it (L.D.); 2 Immunology and Diagnostic Molecular Oncology Unit, Veneto Institute of Oncology IOV-IRCCS, 35128 Padua, Italy; lisa.elefanti@iov.veneto.it; 3 Soft-Tissue, Peritoneum and Melanoma Surgical Oncology Unit, Veneto Institute of Oncology IOV-IRCCS, 35128 Padua, Italy

**Keywords:** familial melanoma, nevi, susceptibility genes, polygenic risk score, risk factors

## Abstract

Genetic susceptibility to nevi may affect the risk of developing melanoma, since common and atypical nevi are the main host risk factors implicated in the development of cutaneous melanoma. Recent genome-wide studies defined a melanoma polygenic risk score based on variants in genes involved in different pathways, including nevogenesis. Moreover, a predisposition to nevi is a hereditary trait that may account for melanoma clustering in some families characterized by cases with a high nevi density. On the other hand, familial melanoma aggregation may be due to a Mendelian inheritance of high/moderate-penetrance pathogenic variants affecting melanoma risk, regardless of the nevus count. Based on current knowledge, this review analyzes the complex interplay between nevi and melanoma predisposition in a familial context. We review familial melanoma, starting from Whiteman’s divergent pathway model to overall melanoma development, distinguishing between nevi-related (cases with a high nevus count and a high polygenic risk score) and nevi-resistant (high/moderate-penetrance variant-carrier cases) familial melanoma. This distinction could better direct future research on genetic factors useful to identify high-risk subjects.

## 1. Introduction

The incidence of melanoma has more than tripled worldwide in the last few decades, with an estimated number of new cases in 2020 of about 325,000 per year [1]. The rates of melanoma vary considerably across countries, reaching the highest values in Australia due to high UV radiation levels combined with a predominantly fair-skinned population [2]. Despite melanoma accounting for less than 5% of all skin cancer types, it is one of the most aggressive and lethal forms if not detected and treated at its early stages [3]. Therefore, the identification of high-risk individuals is of particular concern to improve prevention strategies through specific surveillance protocols. For this reason, one of the greatest challenges is understanding the complex interplay between different melanoma risk factors: the number of common and atypical nevi, phenotypic traits, a positive personal or familial history of melanoma and/or other tumor types, and the UV exposure pattern. In particular, previous research has established that the number of common nevi and the presence of atypical nevi (irregular edges, uneven color, diameter greater than 5 mm) are among the most important independent risk factors for melanoma [4]. For instance, individuals with more than 100 nevi have a melanoma risk about seven times higher than those with less than 15 nevi. Moreover, the presence of one or more atypical nevi increases the risk [5].

Detailed research has been carried out on the genetic evolution from normal melanocytes to melanoma [6,7]. However, the complex mechanisms behind the etiology triggering this malignant transformation are still the subject of study.

An interesting hypothesis of “two divergent pathways” was proposed by Whiteman to try to explain the joint effects of host and environmental factors on melanoma development [8]. One of this model’s pathways considers host factors (e.g., the number and type of nevi, and skin phenotype), the main drivers of the malignant transformation of a melanocyte in individuals with a nevi predisposition, through an initial stimulus of UV radiation. In the second pathway, chronic sun exposure causes both the initiation and progression phases of the disease in people with a low tendency to develop nevi. As a result of this model, two distinct subgroups of patients could be identified: individuals with many nevi and a predisposition to melanocyte proliferation, with melanoma onset particularly in non-sun-exposed areas of the body, such as the trunk and limbs; and patients with few nevi and melanoma arising in anatomic sites exposed to sunlight, such as the head and neck. Nevertheless, the lifetime risk of an individual melanocytic nevus becoming malignant is very low, approximately 0.0005% by age <40 years and 0.003% by age >60 years [9]. Epidemiological and histopathological studies have shown that most melanomas arise de novo, with less than 30% of melanomas arising from pre-existing nevi [10].

Beyond environmental and phenotypic factors, it is estimated that about 5–15% of affected cases have a positive family history for this malignancy [11,12]. This evidence suggests that a genetic predisposition may deeply contribute to the development and progression of the disease [13], especially due to the presence of germline mutations in candidate genes at high, moderate, and low penetrance [14].

By taking into account the extensive research on genetic predisposition favoring nevi and melanoma, this review aims to combine the evidence and proposes a novel interpretation of the complex interplay between nevi and familial melanoma.

## 2. Nevi

Melanocytes are melanin-producing cells that typically reside in the basal layer of the epidermis. When the MAPK pathway is activated, for example by a UV-induced mutation in the *BRAF* (mainly V600E) or *NRAS* genes [15], melanocytes undergo proliferation leading to the development of a nevus, then entering a state of senescence and stabilizing the structure of the nevus for many years. However, senescent melanocytes may resume their proliferation in response to additional stimuli such as further UV exposure, immunosuppression, and pregnancy, changing within the structure of the nevus that may progress to pathological forms until the development of a malignant melanoma [6]. Therefore, UV radiation is an important etiologic factor for nevogenesis, as evidenced by the typical somatic UV-correlated mutation signature (dominated by C > T transitions) detected in most acquired nevi in sun-exposed areas of the body (head and neck) [16]. Congenital nevi, on the other hand, may already be present at birth and developing in the fetus. Similar to acquired nevi, congenital nevi are also characterized by MAPK pathway activation, but mainly due to *NRAS* rather than *BRAF* (usually not V600E) hotspot mutations [17]. Like the acquired nevi located in body regions with low UV exposure (such as the trunk and limbs) [16], congenital nevi commonly present a somatic mutation profile that is not UV-correlated [15]. The congenital nevus usually appears as a solitary lesion [18], while the individual number of acquired nevi may vary from one to over 100. Genetic factors, as well as sun exposure, influence an individual’s susceptibility to developing acquired nevi over their lifetime [19,20,21,22,23]. Indeed, genome-wide association studies (GWAS) identified different loci strongly associated with the nevus count [23,24]. These studies reported single nucleotide polymorphisms (SNPs) on chromosomes 6 (mapping in the *IRF4* gene), 9 (in the *MTAP* and *DOCK8* genes and in the 9q31.2 region), 12 (in the *KITLG* gene), and 22 (in the *PLA2G6* gene) as the most highly predictive of the number of nevi. *IRF4* is a transcription factor implicated in melanocyte pigmentation and proliferation [25]; *MTAP* acts in polyamine metabolism [26]; *DOCK8* regulates signal transduction [27]; *KIT*-ligand (*KITLG*) acts in different biological pathways, including melanocyte development and melanin synthesis [28]; and *PLA2G6* has a crucial role in the regulation of membrane dynamics and homeostasis [29]. Thus, multiple pathways appear to be involved in the susceptibility to nevi, supporting a polygenic model of nevogenesis lacking high-penetrance nevus genes.

Developing many nevi is a hereditary trait [30] following a more complex transmission than the classic Mendelian transmission [31]. It has been postulated that a high nevus count may be correlated to a genetic predisposition for people living in countries with low solar radiation (e.g., the UK) rather than for those in very sunny countries (e.g., Australia), where nevus development may be mainly correlated to sun exposure [32].

Familial aggregation of increased numbers of nevi was first reported in familial atypical multiple mole melanoma (FAMMM) syndrome [33,34], characterized by a family history of melanoma and multiple large nevi, some of which with atypia. However, the dominant genetic traits that predispose to FAMMM syndrome are characterized by germline mutations in melanoma risk loci that are not associated with the nevus count [35].

## 3. Familial Melanoma

It is estimated that about 5% to 15% of melanoma cases occur in individuals with a family history of this malignancy [11,12], suggesting genetic susceptibility. In particular, studies have revealed a multigenic pattern of predisposition, in which both a limited number of rare variants with high/moderate penetrance and quite common variants with low penetrance play a crucial role in a continuous gradient of gene effects that modulate the individual risk of developing melanoma [36] (Table 1).

### 3.1. High-Risk Genes

*CDKN2A* and its binding partner *CDK4* were the first melanoma genes to be identified as melanoma-predisposing genes, though mutations in these loci have only been found in 20–45% of familial melanoma cases [37]. *CDKN2A* (Cyclin-Dependent Kinase Inhibitor 2A) at 9p21 encodes two distinct proteins by alternative splicing (p16INK4A and p14ARF), both involved in cell-cycle regulation [37]. It is mutated in 10% of families with two melanoma cases and up to 30–40% in families with three or more melanoma cases [38], with differences in frequency influenced by geographic regions (from 20% in Australia to 57% in Europe) [14,38,39]. Other variables can influence *CDKN2A* mutation incidence, and one of these is the presence of atypical moles, like in FAMMM syndrome, which has been linked to *CDKN2A*-carrier status, since family relatives with this syndrome seem to have a three-fold risk of carrying a germline variant [40,41]. Then, melanoma patients with nevi in non-sun-exposed skin and dysplastic nevi are more at risk of being mutation carriers [40,41]. In addition, an increased number of affected relatives, a young age at melanoma diagnosis (less than 40 years old) and relatives with multiple melanomas or pancreatic cancer are predictive of being a mutation carrier [38,42,43]. The penetrance of *CDKN2A* mutations; that is, the probability that a mutation carrier develops the disease, is incomplete since it reflects a combination of environmental and genetic factors, such as the geographic region, UV radiation, the melanoma population incidence rate, heritable genetic modifiers (in particular *MC1R*), and age. For this reason, penetrance for mutation carriers at the age of 50 is estimated to be about 13% in Europe, 50% in the US, and 32% in Australia, while it is 58% in Europe, 76% in the US, and 91% in Australia at the age of 80. Indeed, families with melanoma aggregate are predicted to share environmental (UV radiation) as well as phenotypic (e.g., atypical moles) and genetic factors that influence the risk of developing melanoma [44]. In this familial context, *CDKN2A* germline mutations increase the risk of developing melanoma up to 75-fold [12,45] under the influence of shared risk factors such as UV exposure, sunburn, and atypical moles, but not the total number of common nevi or phenotypic traits [43,45].

*CDK4* (Cyclin-Dependent Kinase 4 in 12q14) is an oncogene involved in the same pathway of p16INK4A [37]. Only 18 families with *CDK4* mutation are described worldwide, and only two different mutations involving the same codon (24 in exon 2, in the p16INK4A-binding domain) have been identified [14]. On the basis of available data, and similar to *CDKN2A*, mutations in *CDK4* are also associated with a high number of atypical nevi [12,37], and penetrance is estimated at 74% [14].

Next-generation sequencing has recently allowed the identification of novel rare high-risk variants in genes related to cell-cycle control, such as *BAP1*, and in genes afferent to the telomere maintenance pathway, such as *TERT* and shelterin complex genes *POT1*, *TERF2IP*, and *ACD*.

*BAP1* (BRCA1-Associated Protein 1 in 3p21) encodes a deubiquitinating enzyme involved in different key pathways (regulation of transcription, regulation of cell cycle and growth, response to DNA damage, and chromatin dynamics) [12,37]. Germline *BAP1* mutations increase susceptibility to a wide spectrum of cancers, the so-called *BAP1* tumor predisposition syndrome (*BAP1*-TPDS). This condition is particularly characterized by the presence of BINs (*BAP1*-inactivated nevi) occurring in up to 90% of mutation carriers [12,14]. These lesions are phenotypically distinct from common nevi and suggestive of the presence of germline mutations [14]. It is estimated that 15% of mutation carriers develop melanoma, while the mutation frequency increases up to 28% in families with cutaneous and uveal melanoma [37]. Due to this large and heterogeneous phenotype manifestation, the penetrance of *BAP1* mutation is variable, and probably influenced by unidentified genetic modifiers and/or environmental factors [14,37]. The lifetime risk of mutation carriers developing the above-mentioned cancers is estimated to be around 82.5% [46].

*POT1* (Protection of Telomeres 1) is a shelterin complex gene located in 7q31 that encodes a protein designated for telomere maintenance and protection. Germline mutations in this gene are causative of the *POT1* tumor-predisposition syndrome (*POT1*-TPD), which is characterized by a broad spectrum of malignancies, such as *BAP1*-TPDS [47,48]. *POT1* mutations are found in 2.4% of individuals with familial melanoma [47], and based on available data, their penetrance seems to be very high [14,48].

After *POT1*, *ACD* (ACD Shelterin Complex Subunit and Telomerase Recruitment Factor in 16q22), and *TERF2IP* (TERF2 Interacting Protein in 16q23), shelterin genes were also recently linked to melanoma predisposition and other cancers, even if germline mutations have only been reported in six and four families, respectively [37].

The relevance of telomere biology as a susceptibility pathway for familial melanoma is highlighted by the discovery of a germline mutation in the promoter of the *TERT* gene (c.-57T > G; rs878855297) co-segregating with melanoma in two unrelated families [49,50]. The *TERT* gene (Telomerase Reverse Transcriptase in 5p15) encodes the catalytic subunit of the telomerase enzyme implicated in the maintenance of telomere length, and it is known to be somatically mutated in a variety of tumors, including melanoma [12]. The germline *TERT*-promoter mutation, despite its rarity, seems to be highly penetrant, since almost all carriers have developed early-onset melanoma and/or other tumor types. However, more data is needed for *POT1*, *ACD*, *TERF2IP*, and *TERT* to clarify the penetrance of their mutations and the association with the nevus phenotype [49].

In summary, rare mutations in high-penetrance genes account for melanoma manifestation in approximately 22% of melanoma families (19% for *CDKN2A*, and 3% for the other genes), while the major melanoma genetic factors underlying melanoma in prone families are still unknown [39].

### 3.2. Moderate- to Low-Risk Genes

Moderate- and low-risk genes for melanoma predisposition are mainly involved in pigmentation processes and melanocyte differentiation. Since their alleles are commonly shared by the general population, their effect is not individually sufficient to drive oncogenesis. However, taken together, they can synergistically push the personal risk of melanoma above the critical threshold and overall predispose to the disease [14,51].

The *MITF* (Melanocyte-Inducing Transcription Factor in 3p14) gene regulates pigmentation and the different stages of melanocyte development [37]. A single *MITF* variant (c.G1075A p.E318K; rs149617956) was reported to act as a moderate-penetrance mutation with a three- to five-fold increased risk of melanoma and predisposition to atypical nevi and a high nevus count [12,39,52,53].

*MC1R* (Melanocortin 1 Receptor in 16q24) is a key gene in the pigmentation pathway, since it regulates the ratio between eumelanin (brown pigment that protects against UVR) and pheomelanin (red/yellow pigment) [39]. *MC1R* is highly polymorphic in the general European population [39,54], since these common variants contribute to determining the phenotypic expression of skin and hair color, the tanning response, and sensitivity to UVR’s harmful effects. The *MC1R* variants associated with the red hair color phenotype (RHC) are commonly classified as R alleles and are the most-involved SNPs in melanoma susceptibility. It is estimated that R alleles confer a two-fold risk of developing melanoma in the common population and a three-fold risk in familial clusters [39]. The association between *MC1R* variants and nevus phenotype is still controversial [55,56,57,58]. Even though the role of *MC1R* alleles in phenotypic determination is well known, *MC1R* can affect melanoma susceptibility through both pigmented and non-pigmented pathways [14]. It is also involved in the activation of different DNA repair mechanisms in response to UV-induced DNA damage [14]. Furthermore, some *MC1R* variants can increase the penetrance of a *CDKN2A* mutation when occurring together [39,56], in a complex interplay between *CDKN2A* mutations and individual phenotypes (dysplastic nevi and poor tanning ability) [55,56,58]. Overall, *MC1R* is considered a moderate-risk gene for melanoma development [39].

GWAS population studies identified other different common allelic variants in genes involved in multiple biological pathways, highlighting the complexity of the cutaneous melanoma etiology. SNP mapping in loci associated with nevogenesis (i.e., in *MTAP*, *PLAG2G6*, and *ATM* genes), telomere maintenance (i.e., in *TERT*, *OBFC1*, *PARP1*, and *FTO* genes) and pigmentation (i.e., in *MITF*, *OCA2*, *MC1R*, and *SLC45A2* genes) are associated with cutaneous melanoma susceptibility. While each of these variants alone has a weak effect on melanoma susceptibility (the O.R. for each variant is estimated to be less than two) [14,39,59], their combination, according to a polygenic risk score (PRS) model, may triple the risk of melanoma, and even more so when in combination with the pigmentation and nevus count [60,61].

**Table 1:** The role of the main melanoma-susceptibility genes in melanoma risk.

Genes	Variants’ Prevalence	Variants’ Penetrance	Risk of Melanoma (Relative RISK)	Reference
*CDKN2A*	Low	High	35- to 70-fold	[12,37,43,45]
*CDK4*	Low	High	unknown	[12,14,37]
*BAP1*	Low	High	unknown	[12,14,37]
*POT1*, *ACD*, *TERF2IP*, *TERT*	Low	High	unknown	[12,14,37,47,48,50]
*MITF*	Low	Moderate to low	3- to 5-fold	[12,14,37,39,52,53]
*MC1R*	Moderate	Moderate to low	3-fold	[39,54,55,56,58]
*DOCK8*, *KITLG*, *OCA2*, *MTAP*, *PLA2G6*, *SLC45A2*, *IRF4*, *OBFC1*, *FTO*, *PARP1*, *and others*	High	Low	>3-fold *	[14,39,60,61]

* In the polygenic risk score model.

Taken together, low-prevalence variants in high-penetrance genes and exposure to UV radiation are the major risk factors for familial melanoma. Differences in the amount of exposure to UV radiation related to geographic latitude and the additive effect of high-prevalence variants in low-penetrance genes may contribute to the wide geographic variation in melanoma incidence, also in a non-familial context.

## 4. Nevi and Familial Melanoma

Melanocytic nevi, a positive family history of melanoma, and high-penetrance variants are known important risk factors for melanoma, and the knowledge of their interaction is useful for the definition of overall melanoma risk. Overall, in the general population, epidemiological studies reported that a high number of nevi (>100) increases the risk of melanoma more than a positive family history (RR = 6.89; 95% CI: 4.63–10.25 vs. 1.74; 95% CI: 1.41–2.14, respectively) [5,13] and more so than when harboring a constitutive pathogenic variant in the high-penetrance *CDKN2A* gene (RR = 4.3; 95% CI: 2.4–7.7) [43]. However, *CDKN2A*-mutation carriers in melanoma-prone families have a higher risk of melanoma than *CDKN2A*-mutation carriers in the general population [62], increasing the risk of melanoma by up to 75-fold [12,45]. Moreover, the risk of melanoma in melanoma-prone families is less influenced by the total nevus count than in cases unselected by family history, and it is influenced even less when these families are characterized by a germline high-penetrance variant [45]. A high nevus density does not, therefore, seem to be a melanoma risk factor for *CDKN2A*-mutation carriers [45], as confirmed by the observation of a high nevus count in *CDKN2A* carriers without melanoma diagnosis [40] and few nevi in a number of *CDKN2A*-mutated melanoma patients [63]. A different scenario materializes for atypical nevi, which may increase the risk of melanoma by up to 10-fold when presenting more than five atypical nevi per body [5]. Atypical nevi also act as independent risk markers overall, regardless of family history and pathogenic mutation status; however, unlike common melanocytic nevi, atypical nevi contribute to the development of melanoma synergistically with the *CDKN2A*-mutation carriage, as represented in the FAMMM syndrome [11]. Therefore, *CDKN2A* germline mutations appear to be more correlated with the atypical, rather than with the common, nevus count [40]. It has long been known that the atypical nevus count correlates with the common nevus count [64], and more recently, the role of the *CDKN2A* locus in the development of histological atypia in nevi during melanoma progression was proposed [6]. However, it is important to note that not all atypical nevi progress to melanoma [65]; additional stimuli, such as UV exposure and/or inherent genetic traits, are probably necessary for their malignant transformation [66]. While the total nevus count does not modify melanoma risk resulting from the pathogenic germline mutation status, recent meta-analysis GWAS studies identified several common SNPs that, when combined in a polygenic model, modified the risk of melanoma by acting through nevus development and beyond [23,59]. Interestingly, a meta-analysis GWAS study [60] using 204 SNPs built a PRS by which an Italian population cohort may be stratified into high- and low-melanoma-risk subjects according to the phototype (eye/hair/skin color) and nevus count (<50 or 50+ nevi). The melanoma absolute risk increased for men with a high nevus count and light phototype from about 6% to 12%, depending on their genetic profile, corresponding to the lowest or highest PRS value, respectively. On the other hand, for men with a low nevus count and dark phototype, the PRS affected the absolute risk of melanoma to a much lesser extent, always remaining below 1% with any genetic-risk profile. For subjects with the same medium phototype and the same genetic-risk profile (either low or high PRS value), the absolute risk of melanoma increased by almost three-fold in the presence of a high nevus count compared with a low nevus count. The PRS significantly correlates with the nevus count to act synergistically on the risk of melanoma [60]. Most of these studies on the role of PRS on melanoma susceptibility are population-based [23,59,60,61], and overall account for the individual risk of melanoma. Law et al. recently evaluated the burden of polygenic-risk variants in melanoma-prone families, highlighting a significantly higher PRS in both affected and non-affected members compared to melanoma cases without a family history and healthy controls [36]. Therefore, predisposition to familial melanoma may be enriched for polygenic risk in addition to high/moderate-penetrance variants. Interestingly, melanoma families with high-penetrance mutations had a significantly lower polygenic score than families without mutations [67]. Law et al. hypothesized that melanoma families with a low PRS might be more likely to have germline high penetrance mutations, even though they only found one family harboring a *CDKN2A* pathogenic variant after sequencing the entire genome of 21 families with a low PRS [36].

Taken together, these results provide important insights into the high heterogeneity of melanoma predisposition, leading to the possible identification of two main scenarios in the familial melanoma context. A first subgroup of nevi-resistant melanoma families harboring high/moderate-penetrance variants could be discerned, wherein the risk of melanoma is weakly modified by the polygenic risk score and number of nevi; and a second subgroup of nevi-related melanoma families, characterized by a high polygenic-risk score influencing melanoma risk according to nevus density and the lack of high/moderate-penetrance variants. Since other factors may be involved, we do not expect to observe a clear distinction between these two types of familial melanoma. For instance, in the FAMMM syndrome, which is characterized by the presence of multiple atypical and common nevi, both *CDKN2A* pathogenic variants and the nevus count affect the risk of melanoma in a synergistic manner.

## 5. Conclusions and Future Prospects

Cutaneous melanoma has a complex etiology due to the synergistic interaction of different risk factors. The association between UV-radiation exposure and melanocytic nevi represents the major player in this setting. High UV exposure may contribute to both nevi development and their malignant transformation into melanoma. Even if nevus and melanoma appear to be two closely-related entities [68], cutaneous melanoma may develop independently of the presence of nevi. Indeed, in the majority of patients, melanoma develops as a de novo lesion on healthy skin and only about a third of cases develop on a pre-existing nevus. Moreover, there is no evidence that the nevus count affects the risk of melanoma in carriers of high/moderate-penetrance variants [45], the role of which in nevi development is still debated [40]. Therefore, we define nevi-resistant familial melanoma as the condition that occurs in melanoma-prone families with high/moderate-penetrance mutations. On the other hand, a constitutive genetic background, such as the one profiled by common low-penetrance allelic variants that act synergistically on different biological pathways (including nevogenesis), increases the risk of melanoma, particularly when combined with a high nevus count. Therefore, we define nevi-related familial melanoma as the condition that occurs in families where melanoma clustering may be due to an enrichment for polygenic risk, also favoring nevus development [36].

The distinction we are proposing here stems from the Whiteman et al. [8] model of two divergent pathways to cutaneous melanoma. Sporadic melanoma can occur in individuals without a nevi predisposition as a result of chronic exposure to solar radiation. On the other hand, a melanoma pathway based on host factors may account for familial melanoma, and should be further differentiated into nevi-resistant and nevi-related familial melanoma, according to genetic background. Under the classical Mendelian inheritance, nevi-resistant familial melanoma is characterized by a higher number of affected members and the onset of melanoma at an earlier age, whereas disease transmission in nevi-related familial melanoma follows a more complex inheritance. In this last context, we also expect to observe a high frequency of melanomas arising from pre-existing nevi in anatomic non-sun-exposed areas (Table 2).

These two subgroups of familial melanoma appear to overlap in the FAMMM syndrome in which high-penetrance variants, nevus count, and sun exposure synergistically act on the risk of melanoma.

Future studies are necessary to better define the features of these two proposed types of familial melanoma. In our opinion, differentiating between the two forms of familial melanoma could be useful to better direct research on new rare high/moderate-penetrance melanoma variants (in nevi-resistant familial melanoma) and the new polygenic profiles of common low-penetrance allelic variants (in nevi-related familial melanoma). The common goal in both situations is to improve the identification of subjects at high risk of developing cutaneous melanoma.

## Figures and Tables

**Table 2:** Features mostly represented in nevi-resistant and nevi-related familial melanoma.

	Familial Melanoma	
	Nevi-Resistant	Nevi-Related
High/Moderate-penetrance variant frequency	+	
Polygenic Risk Scores		+
Nevus count		+
High inheritance	+	
Nevus-associated melanoma occurrences		+
Melanomas in anatomic non-sun-exposed areas		+
Early onset of melanoma	+	
